# Barriers That Interfere with Access to Tuberculosis Diagnosis and Treatment across Countries Globally: A Systematic Review

**DOI:** 10.1021/acsinfecdis.4c00466

**Published:** 2024-07-18

**Authors:** Titilade Kehinde Ayandeyi Teibo, Rubia Laine de Paula Andrade, Rander Junior Rosa, Paula Daniella de Abreu, Oluwaseyi Ademo Olayemi, Yan Mathias Alves, Reginaldo Bazon Vaz Tavares, Fernanda Bruzadelli Paulino da Costa, Heriederson Sávio Dias Moura, Letícia Perticarrara Ferezin, Ariela Fehr Tártaro, Mônica Chiodi Toscano de Campos, Natacha Martins Ribeiro, Thaís Zamboni Berra, Ricardo Alexandre Arcêncio

**Affiliations:** † Department of Maternal-Infant and Public Health Nursing, Ribeirão Preto College of Nursing, University of São Paulo, Ribeirão Preto, São Paulo 14040-092, Brazil; ‡ Department of Physiology, Obafemi Awolowo University, Ile-Ife, Osun State 220282, Nigeria

**Keywords:** Barriers, Diagnosis, Tuberculosis, Social Treatment, Patients, Health professionals, Knowledge, Human Resources, World Health Organization

## Abstract

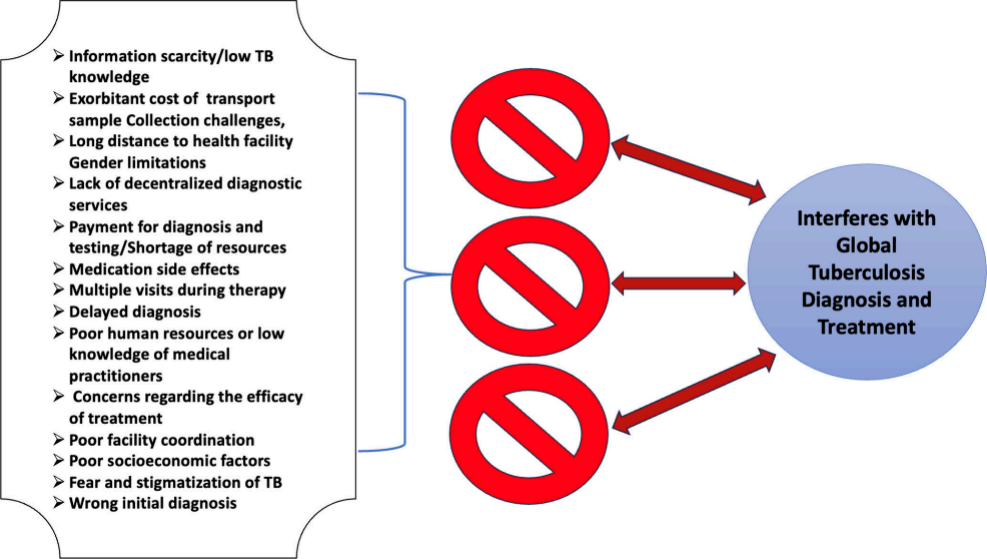

This study evaluated the barriers that interfere with access to diagnosis and treatment of tuberculosis (TB) from the perspective of the patient and health professionals globally. Using the PICo acronym, the question we asked was “What are the barriers that interfere with access to tuberculosis diagnosis and treatment (I) from the perspective of patients and/or health professionals (P) across countries globally (Co)?”. We searched the following databases: EMBASE, Scopus, MEDLINE, Latin American and Caribbean Literature in Health Sciences (LILACS), and Web of Science. On Rayyan, duplicates were removed and extraction was done afterward by two authors independently, followed by a tiebreaker. Using a Critical Appraisal Tool proposed by the Joanna Briggs Institute, the methodological quality of the article was assessed. From 36 published articles, the barriers to tuberculosis diagnosis as obtained from our study include information scarcity/low TB knowledge, exorbitant cost of transport, sample collection challenges, long distance to health facility, gender limitations, lack of decentralized diagnostic services, payment for diagnosis and testing, medication side effects, multiple visits during therapy, delayed diagnosis, poor human resources, low knowledge of medical practitioners, concerns regarding the efficacy of treatment, poor facility coordination, poor socioeconomic factors, fear and stigmatization of TB, and wrong initial diagnosis. The review of studies on TB diagnosis and treatment barriers evidences the diverse barriers to the eradication of tuberculosis. Eliminating these barriers is an onus that lies on policy makers, citizens, and health workers alike, with the joint aim of reducing the global TB burden.

Tuberculosis (TB) programs typically measure their successes by focusing on the number of patients screened, diagnosed, and successfully treated; however, quality of care (or lack of it) is related to health outcomes, and therefore, addressing quality of care in relation to users’ access is a critical investment for TB programs.^1^ This is especially important because poor user access and quality of care can increase the rate of treatment default and failure, which are detrimental to the general health system. Many studies have been conducted through operational research for improving the quality of care addressed to TB patients and their families.^2−4^ The main action of EndTB strategy is eliminating TB as a public health issue by coordinating and focusing disease control through rendering all individuals with active TB disease as noninfectious, ensuring all individuals with latent TB infection remain noninfectious, and also ensuring all individuals without TB infection do not become infected;^5^ therefore, studies devoted to investigate these aspects are valuable to subside the health policies, strategic health actions, and surveillance.

The fight against TB has brought about free basic TB diagnostic tests, medicines, and financial support for people with drug-resistant (DR-TB), but sadly, one in two people with TB face diverse barriers in obtaining this succor in their treatment and recovery trajectory. Even though the WHO defines *equity* as the absence of avoidable social, economic, demographic or geographic differences among groups of people,^6^ several populations across the globe are unable to have prompt use of health services which are supposed to be a fundamental right since health is a right for all citizens.^7^

It is important to highlight that widely among the patients who were diagnosed with Tuberculosis and started the treatment only 71% of them concluded the treatment, this is because of some barriers in getting access to health services.^8^ So, this study is justified because it will analyze existing gaps in relation to access and treatment of tuberculosis patients.

All facilities require political commitment to build high-quality health systems that maximize healthcare in each unique setting by consistently providing care that enhances or maintains health. As a result, as the health system adapts to the changing requirements of the populace, it will become one that is respected and trusted by everybody. It becomes necessary to assess the quality of care for tuberculosis treatment.^9^

Our study aimed to identify research studies that point out the barriers that interfere with access to diagnosis and treatment of tuberculosis from the perspective of the patient and/or health professionals globally.

## Methods

The guiding norm for the development of this systematic review protocol is the Joanna Briggs Institute Evidence synthesis manual methodology, which details the Systematic reviews of qualitative evidence,^10^ coupled with the Preferred Reporting Items for Systematic Review and Meta-Analysis recommendations (PRISMA).^11^ This research protocol has been registered with PROSPERO under registration number {CRD42023466261}.

The study was divided into the following six steps: Theme and research question creation; a description of the inclusion and exclusion criterion; description of the data to be extracted from the chosen studies; evaluation of the included study for review; and analysis of the data and information synthesis.^12^

Therefore, the study’s central question “What are the barriers that interfere with access to tuberculosis diagnosis and treatment from the perspective of patients and/or health professionals across countries globally?” was established utilizing the PICo approach: P (Population: Tuberculosis patients and/or healthcare professionals), I (Phenomenon of interest: barriers to access to tuberculosis diagnosis and treatment), AND Co (Context: countries on a global scale).

Afterward, the criteria for inclusion taken into account were: Original qualitative essays that address obstacles to tuberculosis diagnosis and treatment are written in any language. TB patients and medical professionals may make up the study population.

The search for articles was carried out in September 2023 on EMBASE, Scopus, MEDLINE, Latin American and Caribbean Literature in Health Sciences (LILACS), and Web of Science.

To find as many articles as possible on the subject, we integrated restricted and free domains in the search strategy using the Boolean algorithms OR to separate them and AND to associate them. Each database’s unique characteristics were taken into account while designing search strategies (Table 1).

**Table 1:** Article Search Techniques Used in a Systematic Review on Barriers That Interfere with Access to Tuberculosis Diagnosis and Treatment in Countries Globally

Database/Search Strategy^a^
EMBASE: 1,455 publications
#1 ‘access’/exp OR access OR ‘accessibility’/exp OR accessibility OR accessing
#2 ‘access’/exp OR access OR ‘accessibility’/exp OR accessibility OR accessing
#3 ‘treatment’/exp OR treatment OR ‘therapy’/exp OR therapy OR treat OR ‘diagnosis’/exp OR diagnosis OR ‘diagnostic’/exp OR diagnostic OR ‘health services’/exp OR ‘health services’ OR ‘health service’/exp OR ‘health service’ OR ‘health system’/exp OR ‘health system’ OR ‘health care’/exp OR ‘health care’ OR ‘health-care’/exp OR ‘health-care’ OR ‘health facility’/exp OR ‘health facility’ OR ‘health facilities’/exp OR ‘health facilities’
#4 ‘tuberculosis’/exp OR tuberculosis OR ‘tb’/exp OR tb
#5 ‘patients’/exp OR patients OR ‘patient’/exp OR patient OR case OR cases OR ‘inpatients’/exp OR inpatients OR ‘inpatient’/exp OR inpatient OR ‘outpatients’/exp OR outpatients OR ‘outpatient’/exp OR outpatient OR ‘healthcare professionals’ OR ‘healthcare professional’/exp OR ‘healthcare professional’ OR ‘health care professionals’ OR ‘health-care professionals’ OR ‘health-care professional’/exp OR ‘health-care professional’ OR ‘health professionals’ OR ‘health care professional’/exp OR ‘health care professional’ OR ‘caregivers’/exp OR caregivers OR 'caregiver’/exp OR caregiver OR ‘health care practitioners’ OR ‘health care practitioner’/exp OR ‘health care practitioner’ OR ‘health-care practitioners’ OR ‘health-care practitioner’/exp OR ‘health-care practitioner’ OR ‘healthcare practitioners’ OR ‘healthcare practitioner’/exp OR ‘healthcare practitioner’ OR ‘doctor’/exp OR doctor OR ‘physician’/exp OR physician OR ‘physicians’/exp OR physicians OR ‘nurse’/exp OR nurse OR ‘nurses’/exp OR nurses OR ‘nursing’/exp OR nursing
#6 #1 AND #2 AND #3 AND #4 AND #5
#7 #6 AND [embase]/lim
Scopus: 1,112 publications
TITLE-ABS-KEY(Barriers OR barrier OR problems OR problem OR obstacles OR obstacle OR difficulties OR difficulty OR impediments OR impediment OR hurdles OR hurdle) AND TITLE-ABS-KEY(Access OR accessibility OR accessing) AND TITLE-ABS-KEY(treatment OR therapy OR treat OR diagnosis OR diagnostic OR ″health services″ OR ″health service″ OR ″health system″ OR ″health care″ OR ″health-care″ OR ″health facility″ OR ″health facilities″) AND TITLE-ABS-KEY(Tuberculosis OR TB) AND TITLE-ABS-KEY(patients OR patient OR case OR cases OR inpatients OR inpatient OR outpatients OR outpatient OR ″healthcare professionals″ OR ″healthcare professional″ OR ″health care professionals″ OR ″health care professional″ OR ″health-care professionals″ OR ″health-care professional″ OR ″health professionals″ OR ″health care professional″ OR caregivers OR caregiver OR ″health care practitioners″ OR ″health care practitioner″ OR ″health-care practitioners″ OR ″health-care practitioner″ OR ″healthcare practitioners″ OR ″healthcare practitioner″ OR doctor OR doctor OR physician OR physicians OR nurse OR nurses OR nursing)
MEDLINE: 1,080 publications
(″barrier″[All Fields] OR ″barrier s″[All Fields] OR ″barriers″[All Fields] OR (″barrier″[All Fields] OR ″barrier s″[All Fields] OR ″barriers″[All Fields]) OR (″problem″[All Fields] OR ″problem s″[All Fields] OR ″problems″[All Fields]) OR (″problem″[All Fields] OR ″problem s″[All Fields] OR ″problems″[All Fields]) OR (″obstacle″[All Fields] OR ″obstacles″[All Fields]) OR (″obstacle″[All Fields] OR ″obstacles″[All Fields]) OR (″difficulties″[All Fields] OR ″difficulty″[All Fields]) OR (″difficulties″[All Fields] OR ″difficulty″[All Fields]) OR (″impediment″[All Fields] OR ″impediments″[All Fields]) OR (″impediment″[All Fields] OR ″impediments″[All Fields]) OR (″hurdle″[All Fields] OR ″hurdles″[All Fields]) OR (″hurdle″[All Fields] OR ″hurdles″[All Fields])) AND (″access″[All Fields] OR ″accessed″[All Fields] OR ″accesses″[All Fields] OR ″accessibilities″[All Fields] OR ″accessibility″[All Fields] OR ″accessible″[All Fields] OR ″accessing″[All Fields] OR (″access″[All Fields] OR ″accessed″[All Fields] OR ″accesses″[All Fields] OR ″accessibilities″[All Fields] OR ″accessibility″[All Fields] OR ″accessible″[All Fields] OR ″accessing″[All Fields]) OR (″access″[All Fields] OR ″accessed″[All Fields] OR ″accesses″[All Fields] OR ″accessibilities″[All Fields] OR ″accessibility″[All Fields] OR ″accessible″[All Fields] OR ″accessing″[All Fields])) AND (″therapeutics″[MeSH Terms] OR ″therapeutics″[All Fields] OR ″treatments″[All Fields] OR ″therapy″[MeSH Subheading] OR ″therapy″[All Fields] OR ″treatment″[All Fields] OR ″treatment s″[All Fields] OR (″therapeutics″[MeSH Terms] OR ″therapeutics″[All Fields] OR ″therapies″[All Fields] OR ″therapy″[MeSH Subheading] OR ″therapy″[All Fields] OR ″therapy s″[All Fields] OR ″therapys″[All Fields]) OR (″therapy″[MeSH Subheading] OR ″therapy″[All Fields] OR ″treat″[All Fields] OR ″treating″[All Fields] OR ″treated″[All Fields] OR ″treats″[All Fields]) OR (″diagnosable″[All Fields] OR ″diagnosi″[All Fields] OR ″diagnosis″[MeSH Terms] OR ″diagnosis″[All Fields] OR ″diagnose″[All Fields] OR ″diagnosed″[All Fields] OR ″diagnoses″[All Fields] OR ″diagnosing″[All Fields] OR ″diagnosis″[MeSH Subheading]) OR (″diagnosis″[MeSH Terms] OR ″diagnosis″[All Fields] OR ″diagnostic″[All Fields] OR ″diagnostical″[All Fields] OR ″diagnostically″[All Fields] OR ″diagnostics″[All Fields]) OR ″health services″[All Fields] OR ″health service″[All Fields] OR ″health system″[All Fields] OR ″health-care″[All Fields] OR ″health-care″[All Fields] OR ″health facility″[All Fields] OR ″health facilities″[All Fields]) AND (″tuberculosi″[All Fields] OR ″tuberculosis″[MeSH Terms] OR ″tuberculosis″[All Fields] OR ″tuberculoses″[All Fields] OR ″tuberculosis s″[All Fields] OR ″TB″[All Fields]) AND (″patient s″[All Fields] OR ″patients″[MeSH Terms] OR ″patients″[All Fields] OR ″patient″[All Fields] OR ″patients s″[All Fields] OR (″patient s″[All Fields] OR ″patients″[MeSH Terms] OR ″patients″[All Fields] OR ″patient″[All Fields] OR ″patients s″[All Fields]) OR (″ieee int conf automation sci eng case″[Journal] OR ″case phila″[Journal] OR ″case″[All Fields]) OR (″cases public health commun mark″[Journal] OR ″cases″[All Fields]) OR (″inpatient s″[All Fields] OR ″inpatients″[MeSH Terms] OR ″inpatients″[All Fields] OR ″inpatient″[All Fields]) OR (″inpatient s″[All Fields] OR ″inpatients″[MeSH Terms] OR ″inpatients″[All Fields] OR ″inpatient″[All Fields]) OR (″outpatient s″[All Fields] OR ″outpatients″[MeSH Terms] OR ″outpatients″[All Fields] OR ″outpatient″[All Fields]) OR (″outpatient s″[All Fields] OR ″outpatients″[MeSH Terms] OR ″outpatients″[All Fields] OR ″outpatient″[All Fields]) OR ″healthcare professionals″[All Fields] OR ″healthcare professional″[All Fields] OR ″health-care professionals″[All Fields] OR ″health-care professional″[All Fields] OR ″health-care professionals″[All Fields] OR ″health-care professional″[All Fields] OR ″health professionals″[All Fields] OR ″health-care professional″[All Fields] OR (″caregiver s″[All Fields] OR ″caregivers″[MeSH Terms] OR ″caregivers″[All Fields] OR ″caregiver″[All Fields] OR ″caregiving″[All Fields]) OR (″caregiver s″[All Fields] OR ″caregivers″[MeSH Terms] OR ″caregivers″[All Fields] OR ″caregiver″[All Fields] OR ″caregiving″[All Fields]) OR ″health-care practitioners″[All Fields] OR ″health-care practitioner″[All Fields] OR ″health-care practitioners″[All Fields] OR ″health-care practitioner″[All Fields] OR ″healthcare practitioners″[All Fields] OR ″healthcare practitioner″[All Fields] OR (″doctor s″[All Fields] OR ″doctoral″[All Fields] OR ″doctorally″[All Fields] OR ″doctorate″[All Fields] OR ″doctorates″[All Fields] OR ″doctoring″[All Fields] OR ″physicians″[MeSH Terms] OR ″physicians″[All Fields] OR ″doctor″[All Fields] OR ″doctors″[All Fields]) OR (″doctor s″[All Fields] OR ″doctoral″[All Fields] OR ″doctorally″[All Fields] OR ″doctorate″[All Fields] OR ″doctorates″[All Fields] OR ″doctoring″[All Fields] OR ″physicians″[MeSH Terms] OR ″physicians″[All Fields] OR ″doctor″[All Fields] OR ″doctors″[All Fields]) OR (″physician s″[All Fields] OR ″physicians″[MeSH Terms] OR ″physicians″[All Fields] OR ″physician″[All Fields] OR ″physicians s″[All Fields]) OR (″physician s″[All Fields] OR ″physicians″[MeSH Terms] OR ″physicians″[All Fields] OR ″physician″[All Fields] OR ″physicians s″[All Fields]) OR (″nurse s″[All Fields] OR ″nurses″[MeSH Terms] OR ″nurses″[All Fields] OR ″nurse″[All Fields] OR ″nurses s″[All Fields]) OR (″nurse s″[All Fields] OR ″nurses″[MeSH Terms] OR ″nurses″[All Fields] OR ″nurse″[All Fields] OR ″nurses s″[All Fields]) OR (″nursing″[MeSH Terms] OR ″nursing″[All Fields] OR ″nursings″[All Fields] OR ″nursing″[MeSH Subheading] OR ″nursing s″[All Fields]))
LILACS: 122 publications
(barriers OR barrier OR problems OR problem OR obstacles OR obstacle OR difficulties OR difficulty OR impediments OR impediment OR hurdles OR hurdle OR barreiras OR barreira OR problemas OR problema OR obstáculos OR obstáculo OR dificuldades OR dificuldade OR impedimentos OR impedimento OR barreras OR barrera OR dificultades OR dificultad OR impedimentos OR impedimento) AND (access OR accessibility OR accessing OR acesso OR acessibilidade OR acceso OR accesibilidad) AND (tuberculosis OR tb OR tuberculose) AND (patients OR patient OR case OR cases OR inpatients OR inpatient OR outpatients OR outpatient OR ″healthcare professionals″ OR ″healthcare professional″ OR ″health care professionals″ OR ″health care professional″ OR ″health-care professionals″ OR ″health-care professional″ OR ″health professionals″ OR ″health care professional″ OR caregivers OR caregiver OR ″health care practitioners″ OR ″health care practitioner″ OR ″health-care practitioners″ OR ″health-care practitioner″ OR ″healthcare practitioners″ OR ″healthcare practitioner″ OR doctor OR doctor OR physician OR physicians OR nurse OR nurses OR nursing OR pacientes OR paciente OR caso OR casos OR “profissionais de saúde” OR “profissional de saúde” OR médico OR médicos OR enfermeira OR enfermeiras OR enfermagem OR “profesionales de la salud” OR cuidadores OR cuidador OR médico OR médicos OR enfermeira OR enfermeiras OR enfermeiro OR enfermeiros OR enfermagem) AND (treatment OR therapy OR treat OR diagnosis OR diagnostic OR ″health services″ OR ″health service″ OR ″health system″ OR ″health care″ OR ″health-care″ OR ″health facility″ OR ″health facilities″ tratamento OR terapia OR tratamento OR terapia OR diagnóstico OR diagnóstico OR nfermeira OR ″serviços de saúde″ OR ″serviço de saúde″ OR ″cuidados de saúde″ OR ″cuidados de saúde″ OR ″estabelecimento de saúde″ OR ″instalações de saúde″ OR ″servicios de salud″ OR ″servicio de salud″ OR ″cuidado de la salud″ OR ″centro de salud″ OR ″establecimiento de salud″) AND (db:(″LILACS″))
Web of Science: 683 publications
Barriers OR barrier OR problems OR problem OR obstacles OR obstacle OR difficulties OR difficulty OR impediments OR impediment OR hurdles OR hurdle (Topic) and Access OR accessibility OR accessing (Topic) and treatment OR therapy OR treat OR diagnosis OR diagnostic OR ″health services″ OR ″health service″ OR ″health system″ OR ″health care″ OR ″health-care″ OR ″health facility″ OR ″health facilities″ (Topic) and Tuberculosis OR TB (Topic) and patients OR patient OR case OR cases OR inpatients OR inpatient OR outpatients OR outpatient OR ″healthcare professionals″ OR ″healthcare professional″ OR ″health care professionals″ OR ″health care professional″ OR ″health-care professionals″ OR ″health-care professional″ OR ″health professionals″ OR ″health care professional″ OR caregivers OR caregiver OR ″health care practitioners″ OR ″health care practitioner″ OR ″health-care practitioners″ OR ″health-care practitioner″ OR ″healthcare practitioners″ OR ″healthcare practitioner″ OR doctor OR doctor OR physician OR physicians OR nurse OR nurses OR nursing (Topic)

a Source: authors.

The studies were obtained from the databases and then moved to the Rayyan QCRI portal^13^ for the full elimination of duplicate studies. Two independent researchers subsequently reviewed the full titles and abstracts of the studies, with a third evaluator included where there had been disagreement or ambiguity between the first two. The comprehensive reading of the papers that were selected for this initial step made it easier to assess their applicability for inclusion in the review. We present the study selection process using a flowchart as recommended by the Preferred Reporting Items for Systematic Reviews and MetaAnalyses.^11^

After that, we extracted data using a form that we had modified from ref (10) and included the following variables.: Author, Year, and Journal of publication; and Description of the study (Method, Phenomenon of interest (objective), Study site, Participants, Data analysis, Results, Conclusions, and Comments).

The Joanna Briggs Institute’s criteria for evaluating qualitative research were used to assess the quality of methodology of the papers that were part of the review.^10^

Evidence synthesis involves the aggregation/synthesis of findings to generate a set of statements that represent the information collated, through assembling the findings and categorizing these findings on the basis of similarity in meaning. After that, a single thorough set of synthesis results was created from them, which served as the foundation for an evidence-based discussion. Only credible findings were included in the set. The information obtained was pooled together for discussion.

## Results

**Figure 1:**
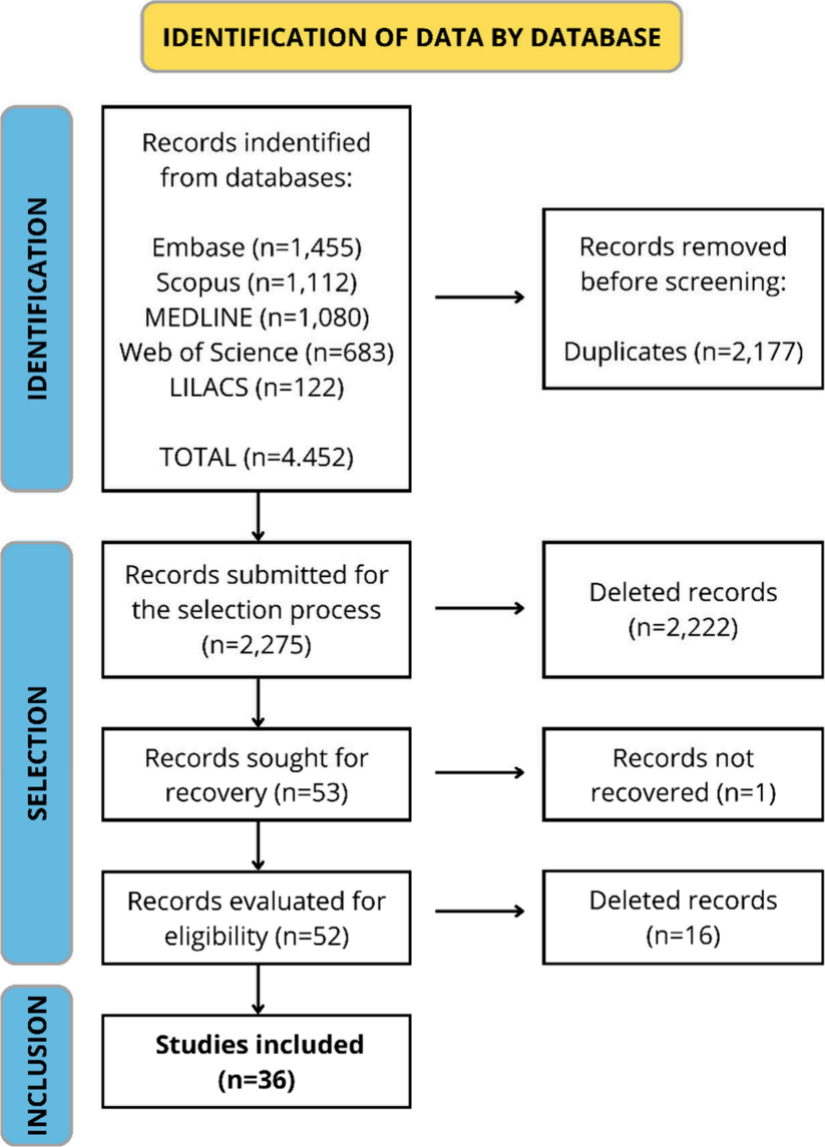
Flowchart of the article selection process of the systematic review of the barriers that interfere with access to tuberculosis diagnosis and treatment across countries globally. Source: Remodeled from ref (11).

A total of 4,452 publications were identified after the searches on the databases. Of them, 2,177 were excluded because they were duplicated and 2,222 after reading their title and abstracts and one that was not recovered. The remaining 52 publications were read fully and 16 were excluded, so 36 studies were included in this review which has 2,447 participants cut across TB patients, caregivers, healthcare providers, and data officers (Figure 1).

**Figure 2:**
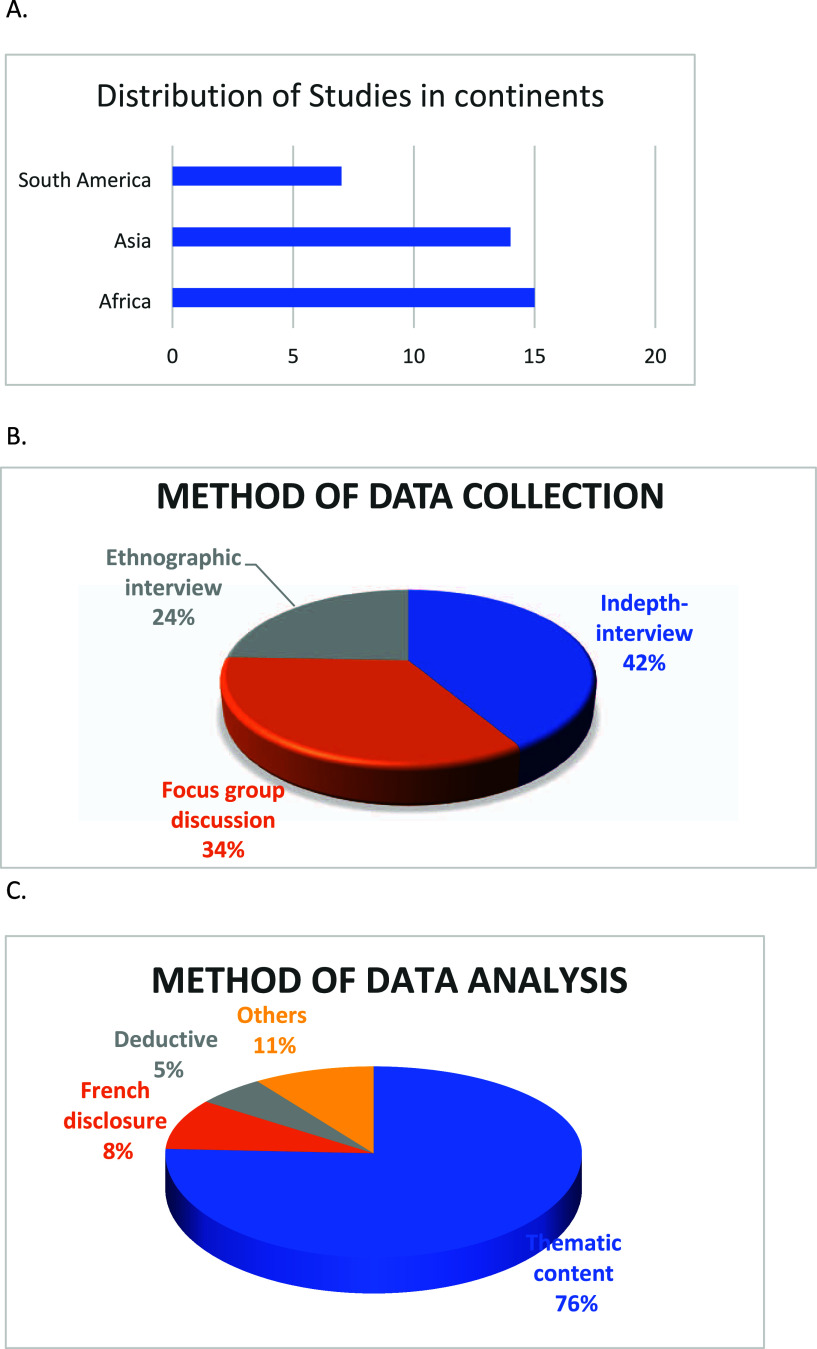
Graphical representation of the descriptions of the studies used. A. Distribution of studies in continents. B. Method of data collection. C. Method of data analysis.

The description of the studies is described in detail in Table 2 where the continent of Africa has 15/36 (42%), Asia 14/36 (39%), and South America 15/36 (19%) and summarized in Figure 2A.

**Table 2:** Features of the Publications That Were Part of the Systematic Review of the Barriers That Interfere with Access to Tuberculosis Diagnosis and Treatment Across Countries Globally

S/N	Ref	Objective	Method	Study Site	Participants	Data Analysis	Results
1.	(14)	Determine which programmatic obstacles prevent patients with coinfection with HIV and tuberculosis from receiving comprehensive care.	In-depth interviews	Peru	16 health providers, 4 patients, and 2 officials	Thematic content analysis	A lack of coordination between HIV and tuberculosis teams, managing HIV and tuberculosis patients separately at different stages of care, inadequate funding, a shortage of skilled staff, and the lack of an information system were the obstacles that were found.
2.	(15)	To comprehend the obstacles that exist in receiving a TB diagnosis and completing treatment in Lesotho.	In-depth interviews and focus group discussions	Lesotho	24 patients, 15 health care workers, and 14 nurses	Thematic content analysis	Challenges during sample collection, a lack of decentralized diagnostic services, and socioeconomic reasons like food hardship and high patient mobility for job searches were the main obstacles to testing and treatment completion.
3.	(16)	To investigate what favors and what hinders people in Xigaze, China, from obtaining and staying in touch with TB care services.	In-depth interviews	China	23 TB patients	Thematic content analysis	Patients reported complicated care paths that frequently required numerous hospital visits, as well as limited awareness of and an indifferent attitude toward tuberculosis. They had trouble physically accessing care, and they had to pay for tests, diagnostics, and transportation out of pocket. Concerns regarding the efficacy of treatments and the negative effects of medications were obstacles to continuing care.
4.	(17)	To evaluate women’s access to healthcare and the general public’s knowledge of tuberculosis.	Focus group discussions and semistructured interview	Pakistan	36 women	Deductive analysis based on the SEM (socio ecological model) and inductive analysis	Access to healthcare for women is hampered by a number of factors, including low autonomy in household financial decision-making, disapproval of unassisted travel, long travel distances, a lack of spending priority on women’s health, and an inadequate number of female health providers. This number is even higher for younger women.
5.	(18)	To examine the narratives of individuals diagnosed with multidrug-resistant tuberculosis, their understanding of how they came to have this illness, and the obstacles they faced when trying to get treatment during the COVID-19 pandemic in a priority city in Brazil.	Semistructured interview	Brazil	7 patients who are undergoing treatment for MDR-TB	French Discourse Analysis	COVID-19 was a significant obstacle for people who needed medical attention. When it came to going back to their doctor’s appointments, many patients expressed fear, nervousness, and anxiety.
6.	(6)	To determine the causes of Nigeria’s low DR-TB case detection and treatment rates.	Documentation review of employee handbooks and guidelines and semistructured interviews	Nigeria	127 TB patients, their treatment supporter, and providers	Thematic content analysis	Unequal patient sociodemographic groups’ access to DR-TB care. Certain patients had more trouble getting care because of their gender, age, occupation, educational background, or religion. Access was probably hindered by restricted coverage and the lack of protection for patients’ access rights as well as considerations in the treatment guidelines and worker manuals.
7.	(19)	To investigate, from the perspective of the healthcare professionals executing TB care, the obstacles and enablers to bidirectional screening.	In-depth interviews	Ghana	23 healthcare workers	Thematic content analysis	Obstacles included skewed funding for screening, fear and stigmatization of tuberculosis, poor teamwork between TB and DM units, and delays in screening.
8.	(7)	To describe the socioeconomic impediments and enablers to TB service access in Nepal	Semistructured focus group discussions	Nepal	14 patients with TB, of which 7 had multidrug resistant TB; 6 community leaders, 7 grass-roots community organizations, and 12 TB health professionals	Thematic content analysis	Socioeconomic hurdles to getting TB services include income loss, stigma, high food and transportation costs, and a lack of awareness and activism around TB.
9.	(9)	Evaluation of Mozambique’s DS-TB, HIV/TB, and MDR-TB service quality, as well as the difficulties in successfully preventing, diagnosing, and treating TB.	Focus group discussions	Mozambique	51 TB patients	Thematic content analysis	Many obstacles were noted by the respondents, such as long wait times at medical facilities, stigma associated with diagnosis and treatment, delayed diagnosis, lack of nutritional support for TB patients, lack of a comprehensive psychosocial support program, and low community awareness of TB or multidrug-resistant TB.
10.	(20)	To comprehend the difficulties faced by TB patients from Myanmar who reside near the border between Thailand and Myanmar in getting access to a TB clinic in a Thai hospital.	In-depth interviews	Thailand	22 TB patients, patients’ relatives and health care providers	Thematic content analysis	Issues with language and finances, lengthy wait times and little information about the infection, excessive workload, and the inappropriateness of some techniques or technologies, Thailand’s national universal insurance program does not subsidize foreign TB patients, and it may occasionally be necessary to return TB patients to their home nation.
11.	(21)	To investigate impediments to accessing TB health care, including confirmatory diagnosis, treatment adherence, and recurrence of pulmonary tuberculosis, from the perspectives of patients, physicians, and policymakers.	Semistructured in-depth interview	Iran	33 TB patients	Thematic content analysis	Patients’ poor knowledge of TB symptoms, physicians’ failure to screen for TB among at-risk patients, similar symptoms between TB and other lung diseases, low sensitivity of TB diagnostic tests, incomplete case finding and contact-tracing, stigma associated with TB, and patients’ poor adherence to long-term TB treatment.
12.	(22)	To investigate obstacles to accessing TB care and information gaps by gender and critical demographics.	Consultative meetings, comprehensive desk review, in-depth interviews and focus group discussion	Cambodia	39 people living with HIV, 31 TB survivors, 41 aged 55 and older, 30 close contacts of people with smear-positive TB, five people with diabetes, 8 prisoners, 20 people who use drugs, and 32 service providers	Thematic content analysis	Lack of knowledge and awareness around tuberculosis
							Insufficient funds and time; Gender-specific impediments in access to TB services
							Lack of resources in health centers to support proper care of tuberculosis
13.	(23)	To describe social and behavioral health factors for successful tuberculosis services and management from the standpoint of miners/ex-miners, health care personnel, and policymakers/managers.	Ethnographic interviews	South Africa	30 miners/ex-miners, 13 family/community members, 14 health care providers, and 47 local policy makers/managers	Iterative analysis	Miners and ex-miners felt that health-care delivery systems did not meet their needs. Many had suffered needless mental and physical suffering because of poor health education on tuberculosis, low engagement in their own treatment, a lack of trust in medical professionals, and a system that overlooked their experience. The stigma and anxiety associated with tuberculosis lead to denial of symptoms and delays in seeking care. Health care professionals and policymakers/managers were deterred by system limits in providing optimal treatment.
						Corbin and Strauss grounded theory procedure	
14.	(24)	To identify challenges and enablers of TB contact research during its implementation in Kampala, Uganda.	Focus group discussions and interviews	Kampala, Uganda	37 nurses, 5 medical officers, 7 clinical officers, 5 lab technicians, 2 counselors, 3 pharmacy technicians, 1 data officer, and 1 multiclinic supervisor	COM-B model - Behavior Change Wheel.	Stigma, poor understanding of tuberculosis among contacts, insufficient time and space in clinics for counseling, mistrust of health-care workers among index patients and contacts, and high travel expenses for LHWs and contacts.
15.	(25)	To explore access to TB, TB/HIV, and multidrug-resistant tuberculosis (MDR-TB) therapy, focusing on barriers to care and enabling variables.	Informant interviews and focus group discussions	Thailand	12 key informants (public health officials and TB treatment providers, migrants and refugees who were receiving TB, TB/HIV and MDR-TB treatment, non-TB patients)	Thematic content analysis	Both migrants and refugees only have access to and eligibility for treatment based on their legal status. Migrants seeking treatment for tuberculosis face financial and nonfinancial impediments to travel and treatment. Important health system elements that impact accessibility include the language of health care, access to free or low-cost therapy, and psychological support.
16.	(26)	To comprehend the factors influencing the ease of TB diagnosis. from the perspective of medical experts.	Semistructured interviews	Paraná and Brazil	20 nurses and 10 doctors	Thematic content analysis	Access to the diagnosis of TB is a difficult deferral of the tests.
17.	(27)	To determine obstacles to pediatric TB diagnosis in Lima, Peru.	Focus group discussions and in-depth interviews	Peru	53 primary care providers, community health workers, and parents and/or guardians of pediatric TB patients	Inductive thematic Analysis	Lack of knowledge and stigma in the community, poor follow-up, restricted availability of diagnostic testing, staff at health centers with insufficient training, and a scarcity of providers. The difficulty of collecting sputum and the limited sensitivity of culture and smear microscopy.
18.	(28)	To examine the sociocultural, geographic, economic, and health system barriers that prevent individuals in Yemen from undergoing and completing the TB testing procedure.	In-depth- interviews and focus group discussions	Yemen	497 TB suspects	Thematic content analysis	The majority of patients had low literacy and were underprivileged, had left rural regions to travel for treatment. Other barriers to TB treatment were distance from home, high social stigma, expense of the clinic and transportation (increased by companions), and ignorance of the diagnostic procedure. Patients have no idea that tuberculosis treatment is free. Referrals to the private sector deterred patients from returning as well.
19.	(29)	To give a contextualized knowledge of how individuals with disabilities might obtain TB care in a particular southern Malawi district.	Semistructured interviews and site observations	Malawi	47 persons with disability, 11 parents/guardians of youths with disability, eight health workers, four community rehabilitation assistants and volunteers, and 19 leaders in the community	Thematic content analysis	Inadequate knowledge and information. Challenges to taking tests. Logistic and operational impediments. Absence of disability-specific policies in the community’s health services
20.	(30)	To examine the obstacles to older people’s TB diagnosis and their access to medical care in João Pessoa, Paraíba, Brazil	Semi structured interviews	Brazil	7 elderly people with TB	French discourse analysis	Family health unit operation hours; delegated duties; home visits without communicant control; wait times for the health service to detect a sickness and for the patient to visit the center many times before receiving a diagnosis.
21.	(31)	To comprehend the obstacles TB patients, face when attempting to access medical treatment.	In-depth interviews	Pakistan	23 TB patients and 15 health personnel	Thematic content analysis	Long distance from health facility, lack of patient awareness, job loss, financial strain, and social stigma
22.	(32)	To outline the difficulties impoverished rural Zambians living with HIV and TB encountered in getting access to ART.	Focus group discussions and semistructured individual interviews	Zambia	14 TB patients and their households	Thematic content analysis	Financial obstacles, societal obstacles. discrimination and the challenge of transparency, barriers seen at health facilities.
23.	(33)	To determine and comprehend the limitations that managers and community care workers (CCWs) perceive in the health systems that affect the execution of joint TB/HIV initiatives, such as PMTCT (prevention of mother-to-child transmission of HIV).	In-depth interviews and focus group discussions	South-Africa	33 health managers and managers of NGOs involved in TB and HIV care, CCWs	Thematic content analysis	The strategy was not implemented with enough consultation, and there was a lack of political will and leadership. Health systems hurdles are mostly associated with constraints connected to organizational culture and structure; management, planning, and power concerns; uneven finance; human resource capability; and regulatory issues, particularly those pertaining to the scope of practice of nurses and CCWs.
24.	(34)	To investigate the factors causing pastoralist TB patients in Ethiopia’s Somali Regional State (SRS) to postpone diagnosis.	Consultation sessions and open interviews	Ethiopia	Seven pastoralist TB patients	Thematic content analysis	Prompt biomedical diagnosis of tuberculosis (TB) among pastoralist TB patients in the Southern Region of Ethiopia was impeded by limited access to professional health care and cultural beliefs that encouraged self-treatment.
25.	(35)	To investigate factors that help and hinder the management of tuberculosis therapy in Addis Ababa, Ethiopia, during the first five months of treatment.	In-depth interviews and focus groups discussions	Ethiopia	44 TB patients, their relatives and health personnel	Systematic text condensation	Employment loss. Routines at health clinics were strict, requiring a lot of time and physical exertion every day. Particularly susceptible to nonadherence were patients who were impoverished as a result of their disease or delayed course and who were unable to improve their social standing and general state of health.
26.	(36)	To comprehend the health-seeking behavior of these individuals as well as the responses of the health systems to their persistent cough in order to determine the variables impacting the delays that both permanent urban residents and migrants experience in acquiring a TB diagnosis in urban China.	Focus group discussions and semistructured interviews	China	20 TB ’suspects’, 17 TB patients and 23 key informants (health managers and health workers)	Thematic content analysis	Inadequate prescription of diagnostic tests and referral to TB clinics by general health practitioners; limited financial ability to pay for care and diagnostic testing; little awareness and poor understanding of tuberculosis (TB) and the TB control program among the general population and TB suspects all serve as obstacles to diagnosis.
27.	(37)	To investigate disparities between genders in behavior related to seeking care, access to treatment, and understanding and views on TB.	Semistructured questionnaires	The Gambia	15 government health and 30 TB patients	Thematic content analysis	Due to time restrictions, higher secrecy, and stronger traditional values, women were more likely to employ traditional healers. All patients, regardless of gender, admitted to having trouble paying for the transportation expenses required to get to the clinic. Patients’ and healthcare professionals’ unfavorable opinions of TB were brought to light. It was commonly claimed that stigma and ignorance about TB were worse among female patients.
28.	(38)	To determine what obstacles and enablers exist at the patient and healthcare system levels in Uganda for the start of TB therapy.	In-depth interviews	Uganda	31 patients, 10 health managers and 38 healthcare workers	Thematic analysis	Inadequate documentation of patient addresses, inability to obtain sputum results from the laboratory, and ignorance of the percentage of patients who are not started on tuberculosis therapy Notable obstacles for patients were delayed sputum results turnaround times and insufficient funds for transportation back to medical facilities (physical opportunity); stigma (social opportunity) and inadequate awareness of tuberculosis (psychological competence).
						COM-B > model.	
						Behavior Change Wheel.	
29.	(39)	To comprehend the experiences of patients in Rio de Janeiro State, Brazil, about the challenges they encountered during the diagnosis and treatment of multidrug-resistant TB, as well as the resulting effects.	Semistructured interviews	Brazil	31 patients undergoing treatment for multidrug-resistant tuberculosis	Thematic content analysis	Multidrug-resistant TB takes longer to diagnose and treat in patients; healthcare professionals do not value or pursue the diagnosis of drug-resistant tuberculosis, poor report rates of active case-finding and contact tracking in primary health centers, insufficient treatment for drug-susceptible TB, and patients display a lack of understanding of the illness.
30.	(40)	To determine potential obstacles to TB centered diagnosis the northwest Ethiopian region of East Gojjam Zone.	In-depth interviews and focus-group discussions	Ethiopia	21 TB patients, 6 TB control officers, and 40 health workers	Thematic content analysis	Health facility barriers include health service delay, using only passive TB centered diagnosis strategy, poor health education provision, lack of continuous oversight and timely feedback, and residence in a rural area, low income, poor health literacy, and delayed health-seeking. Health workers’ barriers include a shortage of HWs, limited training access, and low level of knowledge and skills.
31.	(41)	To investigate and contrast the diagnosis and treatment start pathways experienced by MDR-TB patients using Xpert MTB/RIF-based diagnostic methods and GenoType MTBDRplus.	In-depth interviews using a semistructured guide	South Africa	26 TB patients	Deductive and inductive analysis,	Delays may have resulted from patients delaying seeking medical attention and using the private sector, which was partly caused by widespread perceptions of subpar public sector treatment. The inability of healthcare practitioners to test for tuberculosis (TB) during first patient interactions, deviation from testing protocols, unavailability of test findings, and delayed patient recall for positive results.
32.	(42)	To examine the management topics’ discourse on the elderly’s delayed diagnosis of tuberculosis in municipalities in the Curimataú-Paraíba area.	Interviews	Brazil	9 health managers	French discourse analysis.	Delays in seeking medical attention and understanding sickness, ignorance of the condition, bias, obstacles to receiving care, and a lack of confidence in the ability of specialists to recognize possible instances.
33.	(8)	To investigate the variables influencing TB patients’ access to healthcare, diagnosis, and completion of treatment in central and western Nepal.	In-depth interviews and focus group discussions	Nepal	202 participants from communities, private sector health service provider, government health service providers, a traditional health service provider; TB patients and suspected patients	Thematic content analysis	Long distance, bad roads, and travel expenses. In addition, there was a misconception that early detection of tuberculosis was hampered by a lack of equipment, a shortage of educated medical professionals, and sporadic medical staff attendance. The stigma, the rigorous treatment schedule, and the requirement to attend health centers every day for DOTS treatment posed further obstacles to adherence and treatment completion.
34.	(43)	To comprehend the obstacles that migrant TB patients in Shanghai have in receiving care for tuberculosis (TB) following the implementation of the TB-free treatment strategy.	In-depth interviews	China	34 migrant TB patients	Thematic content analysis	The largest obstacles to TB treatment among migrant patients were said to be financial ones. Both prior to and following being diagnosed with tuberculosis, many migrant patients faced exorbitant medical expenses. Patients who were immigrants reported being shunned or fired from their jobs as a result of their TB diagnosis. They also had little awareness of the free TB treatment program.
35.	(44)	To comprehend the obstacles undergraduate students, face in controlling and preventing TB.	In-depth interviews	China	10 leaders and health workers in the health-care department, 12 individuals in the district centers for disease control, and 15 undergraduates with TB	Thematic content analysis	The national TB policy is not well-accepted, infirmaries and district TB control agencies have insufficient staff and operate subparly, and there is insufficient focus on TB prevention. Additionally, there is a lack of collaboration in the identification, monitoring, and treatment of TB-affected students.
36.	(45)	To get an understanding of their viewpoints of the variables influencing the results of patient treatment and to provide possible programming solutions for improving patient care services.	Mixed-methods study and in-depth interviews	Philippines	272 healthcare workers	Thematic content analysis	Inadequate financial and political backing, a shortage of personnel, and a lack of awareness among healthcare professionals on DRTB. More detailed, contextualized, and subtle facets of every significant difficulty were disclosed through interviews. The detailed obstacles related to patients included costs associated with treatment (such as transportation); anxiety about stigma from the community, family, or healthcare professionals; concerns about medication side effects; a lack of family support; the location of the patients’ homes; the facility staff’s limited ability to provide DRTB care because of a shortage of personnel; the lack of funding to support treatment completion (such as transportation allowance and food packages for patients, service vehicles and cell phone costs for facility-level outreach actions); and discrimination against patients with DRTB that was attributed to the staff’s limited knowledge and experiences of treating the patients

The methods of data collection used are In-depth interviews 17 (42%), focus group discussion 14 (34%), and ethnographic interviews 10 (24%) as shown in Figure 2B.

The method of data analysis used the most is thematic content analysis, 28/37 (76%), French disclosure, 3/37 (8%), deductive analysis, 2/37 (5%), others, 4/37 (11%). The study participants included patients, caregivers, healthcare providers, and data officers (Figure 2C).

Synthesis of the types of barriers experienced across the general population worldwide is captured in Table 3.

**Table 3:** Barriers to Tuberculosis Diagnosis and Treatment across Countries Globally Identified in the Systematic Review

Barrier type	Ref
Information scarcity/Low TB knowledge	(14, 16, 7, 9, 20−24, 27−29, 31, 34, 36, 38−40, 42, 43, 4)
Transport cost	(14, 17, 7, 21, 24, 25, 18, 21, 25−29, 32−34, 36)
Sample collection challenges	(15, 6, 19, 7, 20−22, 25, 27, 29, 35, 38, 8, 44)
Long distance to health facility	(3−6, 8, 9, 12, 14, 15, 28, 31, 35−38, 40, 8, 45)
Gender limitation	(17, 6, 22, 37)
Lack of decentralized diagnostic services	(14, 15, 17, 18, 6, 19, 7, 9, 20, 22, 24, 25, 27−30, 33, 34, 36, 40, 8, 44)
Payment for diagnosis and testing/Shortage of resources	(16, 7, 20, 22, 24, 25, 28, 31, 32, 36, 38, 8, 43, 45)
Medication side effect/burden	(15, 6, 19, 7, 20−23, 45)
Multiple visits	(16−18, 20, 21, 24, 25, 27, 30, 31, 35−37, 41, 8)
Delayed diagnosis	(17, 6, 19, 9, 20, 21, 23, 25−27, 30, 32, 34−36, 38−41, 44)
Poor human resources (low knowledge of medical practitioners)	(14, 19, 9, 21, 23, 27, 30, 33, 37−41, 44, 45)
Concerns regarding the efficacy of treatments	(16, 23, 24, 41, 45)
Poor facility coordination	(14, 19, 9, 20−24, 27, 29, 30, 33, 35, 38−40, 8)
Poor socioeconomic factors	(15, 17, 18, 6, 7, 9, 20, 22, 23, 25, 28, 29, 31, 32, 34−36, 38, 40, 43, 45)
Fear and stigmatization of TB	(18, 19, 7, 9, 21, 23, 24, 27, 28, 31, 32, 37, 8, 43, 45)
Wrong initial diagnosis	(21, 28, 36, 39)

Almost all studies achieved academic excellence when they were evaluated. Only in one study^31^ was it not clear that there was congruence between the research methodology and the representation and analysis of data (Table 4).

**Table 4:** Methodological Quality Assessment of Articles Included in the Systematic Review of the Barriers That Interfere with Access to Tuberculosis Diagnosis and Treatment Across Countries Globally^a^^,^^b^

Ref	1. Is there congruence between the stated philosophical perspective and the research methodology?	2. Is there congruence between the research methodology and the research question or objectives?	3. Is there congruence between the research methodology and the methods used to collect the data?	4. Is there congruence between the research methodology and the representation and analysis of data?	5. Is there congruence between the research methodology and the interpretation of results?	6. Is there a statement locating the researcher culturally or theoretically?	7. Is the researcher’s influence on research and vice versa addressed?	8. Are participants and their voices adequately represented?	9. Is the research ethical according to current criteria or, for recent studies, is there evidence of ethical approval by an appropriate body?	10. Do the conclusions drawn in the research report stem from data analysis or interpretation?
(14)	Y	Y	Y	Y	Y	Y	Y	Y	Y	Y
(15)	Y	Y	Y	Y	Y	Y	Y	Y	Y	Y
(16)	Y	Y	Y	Y	Y	Y	Y	Y	Y	Y
(17)	Y	Y	Y	Y	Y	Y	Y	Y	Y	Y
(18)	Y	Y	Y	Y	Y	Y	Y	Y	Y	Y
(6)	Y	Y	Y	Y	Y	Y	Y	Y	Y	Y
(19)	Y	Y	Y	Y	Y	Y	Y	Y	Y	Y
(7)	Y	Y	Y	Y	Y	Y	Y	Y	N	Y
(9)	Y	Y	Y	Y	Y	Y	Y	Y	Y	Y
(20)	Y	Y	Y	Y	Y	Y	Y	Y	Y	Y
(21)	Y	Y	Y	Y	Y	Y	Y	Y	Y	Y
(22)	Y	Y	Y	Y	Y	Y	Y	Y	Y	Y
(23)	Y	Y	Y	Y	Y	Y	Y	Y	Y	Y
(24)	Y	Y	Y	Y	Y	Y	Y	Y	Y	Y
(25)	Y	Y	Y	Y	Y	Y	Y	Y	Y	Y
(26)	Y	Y	Y	Y	Y	Y	Y	Y	Y	Y
(27)	Y	Y	Y	Y	Y	Y	Y	Y	Y	Y
(28)	Y	Y	Y	Y	Y	Y	Y	Y	Y	Y
(29)	Y	Y	Y	Y	Y	Y	Y	Y	Y	Y
(30)	Y	Y	Y	Y	Y	Y	Y	Y	Y	Y
(31)	Y	Y	Y	N	Y	Y	Y	Y	Y	Y
(32)	Y	Y	Y	Y	Y	Y	Y	Y	Y	Y
(33)	Y	Y	Y	Y	Y	Y	Y	Y	Y	Y
(34)	Y	Y	Y	Y	Y	Y	Y	Y	Y	Y
(35)	Y	Y	Y	Y	Y	Y	Y	Y	Y	Y
(36)	Y	Y	Y	Y	Y	Y	Y	Y	Y	Y
(37)	Y	Y	Y	Y	Y	Y	Y	Y	Y	Y
(38)	Y	Y	Y	Y	Y	Y	Y	Y	Y	Y
(39)	Y	Y	Y	Y	Y	Y	Y	Y	Y	Y
(40)	Y	Y	Y	Y	Y	Y	Y	Y	Y	Y
(41)	Y	Y	Y	Y	Y	Y	Y	Y	Y	Y
(42)	Y	Y	Y	Y	Y	Y	Y	Y	Y	Y
(8)	Y	Y	Y	Y	Y	Y	Y	Y	Y	Y
(43)	Y	Y	Y	Y	Y	Y	Y	Y	Y	Y
(44)	Y	Y	Y	Y	Y	Y	Y	Y	Y	Y
(45)	Y	Y	Y	Y	Y	Y	Y	Y	N	Y

a Y means Yes.

b N means NO.

## Discussion

This study using systematic review has collated the various barriers revolving around TB diagnosis and treatment from the literature evidence. These barriers to TB are impudent toward achieving the EndTB goals of the WHO for the year 2030. Notable among them is that the pillar of these barriers is poor socioeconomic status, poor facility coordination, information scarcity about TB knowledge, and fear and stigmatization of TB.

From this study, it is evident that low TB knowledge among the population is the main driver that proffers interference with TB diagnosis and treatment as majority of the people are not knowledgeable enough of what to do when they have some symptoms and how to get proper care and treatment especially in the rural areas. This is evident in the studies in refs (46 and 47) who found that Ethiopian patients’ knowledge on the cause, treatment, and prevention of TB was inadequate in the most critically ill patients than those who were less critically ill. Adequate knowledge about TB signs and symptoms, its transmission, and how patients (should) seek healthcare when infected with TB, knowledge of the cause of TB, and TB treatment and prevention should be made available to the general populace as it lacks elevates greatly the risk of the transmission. The study carried out in ref (48) in India highlighted gaps in the knowledge of the population regarding the risk of TB transmission in overcrowded areas. This calls for the attention of health managers and policy makers to put in place schemes directed toward a robust information dissemination regarding TB.

Facility coordination involves how health services coordinate the collection of samples for testing, diagnosis, treatment, and follow up, and these, when poorly managed, serve to slow down the whole process of TB diagnosis and treatment. Delayed diagnosis impedes information about the status of TB in individuals, hence preventing prompt care and treatment of TB as this increases the spread of the disease as a result. According to the study in ref (49), delayed diagnosis is a complex situation that creates a cascade of prolonged waiting time on the part of patients, and loss of follow-up of patients, as they end up not returning for treatment. Most of them are lost because of incomplete address information. As a result of this, they are left undiagnosed and go on to infect others in the community, thereby increasing the TB burden.^50^ The passive nature of diagnosis in every way unwinds the success of TB control, as evidenced by ref (51), which also found that when diagnosis is delayed or wrongly done, patients and their families do not seek care and TB therapy is altered leading to drug resistance, a condition that is even worse to handle.

Poor human resources in terms of knowledge of medical practitioners aggravate the problem of optimum control of TB. Most medical practitioners, viz. doctors, nurses, lab assistant, technicians, and nursing assistants, lack updated knowledge about evolution in TB diagnosis and as such cannot apply new knowledge and information to enhance TB care. In the study in ref (49), researchers found that some doctors who are not lung specialists consider TB as a diagnosis very late, and lung specialists in the early stages of diagnosis consider chronic lung diseases earlier on. This poverty of human resources is complemented by the fact that there is inadequate patient training and counseling on the necessity of treatment initiation.^52^

The lack of decentralized diagnostic service causes pressure and backlog to be built in major centers, hence preventing access to prompt diagnosis results. The mode of operation of TB programs in Cities and Towns where different phases of treatment are separated between different facilities slows down therapy. Programs are run vertically and separately from one another, and most times, patients need to visit one facility for initial diagnosis, for example, and are later referred to another for imaging, for sputum collection, and so on. Uwimana et al.^33^ found that this physical separation between programs resulted in the loss of patients in the process of referring them from one facility to another. The long queues in each of these facilities are not endurable by most of them, and those from rural areas dread missing return means of transport. This ultimately slows feedback and creates delays in treatment initiation.

The cost of diagnosis and testing is expensive and most times is paid out-of-pocket, and this is a major barrier in verifying if an individual will proceed to specific treatment. Immunocompromised patients, who are more susceptible to TB and live in precarious conditions, when subject to out-of-pocket payment for treatment, are highly affected by the toll of therapy on their limited finances. A study by SINHA et al.,^53^ carried out in India, found that people living with TB in India face exorbitant costs in the course of treatment directly or indirectly. They iterated that even before treatment begins, a lot of cost is being accumulated. Nadjib et al.^54^ also found that significant costs from patients’ viewpoints are incurred due to the high cost of transportation and the high value of productivity loss. Programs like universal healthcare services and favorable health insurance services can reduce the catastrophic cost of TB treatment.^55^

Actively interfering with TB diagnosis and treatment are fear and stigmatization of TB which are associated with improper knowledge of TB and society’s treatment of individuals with the disease. This causes people to cover up symptoms, prevents seeking diagnosis, and also affects the continuity of TB care and treatment. According to ENDO et al.,^45^ patients are not only worried about being stigmatized by other people within the same community but also by their own family members and the least expected group, healthcare staff. A study developed by Chen et al.^56^ found that among TB patients, stigma was pervasive. In TB stigma-related interventions, special attention should be given to female patients and those with mild to severe disease. Furthermore, it is crucial to stress the importance that doctor–patient contact and social support have in lowering the stigma associated with tuberculosis.

Socioeconomic factor is not a new barrier to TB diagnosis and treatment, and several studies^57,50^ have reported this. It has been highlighted several times that people living in poor socioeconomic conditions have more cases of TB, difficulties in testing/diagnosis, as well as treatment and follow up. This is reflected in why transport cost is one of the barriers to TB diagnosis and treatment as patients from rural areas have to visit centralized testing and treatment centers which impact sample collection challenge as they also revisit for treatment and follow-up where most patients as well as caregivers are most times low on finances and are now getting better. The danger is that most times, this can lead to drug-resistant TB as treatment most times is not completed.

Gender limitation in TB treatment is recurrent; it has been reported that women and children are more vulnerable to be treated due to socioeconomic conditions. Wrong initial diagnosis delays the focus on TB as wrong diagnosis hampers prompt TB treatment and care. According to Yi et al.,^22^ the barriers to TB diagnosis and treatment faced by the female participants are the attachment and commitment they have toward taking care of their families. The male participants sought treatment and diagnosis less because of alcohol consumption and overt ego, confining symptoms for a long period. This agrees with the study by Teo et al.,^58^ who found that women are simultaneously committed to many tasks that keep them away from personal healthcare, while men are patriarchal and therefore downplay healthcare. Health awareness tailored toward the specific need of each gender should be carried out, with focus on vulnerable populations.

There is a concern on the side of patients about the efficacy of treatment and also medication side effects, and sometimes, in search of a faster success rate in treatment, patients look for alternative routes of treatment which in most cases is not effective and precise. Also, side effects of medications happen in progress following treatment for TB.

As limitations of our study, the sources of data search did not include gray literature; also, making use of qualitative studies, our study was not elaborated as a meta synthesis of qualitative studies but as a systematic review adapted to dichotomize qualitative research.

## Recommendation and Conclusion

Based on the barriers discussed, here are some recommendations to address the barriers: more awareness about TB care and diagnosis as this can reduce fear and stigmatization; increase resource allocation to enhance TB diagnosis and treatment so as to ensure creation of more centers involved in TB testing, which will greatly improve prompt TB diagnosis and directly impact positively TB treatment, also, retraining of healthcare officials on-the-job so that they have updated knowledge about tuberculosis; government should work in tandem with WHO EndTB strategy, implement, adapt, and evaluate progress toward the 2035 EndTB target. It is necessary to accomplish worldwide milestones and targets for reducing the number of people infected with TB according to the WHO agenda of ending TB globally with a joint effort between all key stakeholders from government to nongovernment organization, industry, and healthcare professionals. These overall efforts will strengthen the system and overcome these barriers of TB diagnosis/testing, treatment, and care globally. The main actions of EndTB strategy are eliminating TB as a public health issue by coordinating and focusing disease-control through rendering all individuals with active TB disease as noninfectious; and ensuring all individuals with latent TB infection remain noninfectious and also ensuring all individuals without TB infection do not become infected, therefore research devoted to investigate these aspects are valuable to subside the health policies, strategic health actions, and surveillance.
